# Correction: Biba et al. A Comparison of Sanger Sequencing and Amplicon-Based Next Generation Sequencing Approaches for the Detection of HIV-1 Drug Resistance Mutations. *Viruses* 2024, *16*, 1465

**DOI:** 10.3390/v17081059

**Published:** 2025-07-29

**Authors:** Camilla Biba, Lia Fiaschi, Ilenia Varasi, Chiara Paletti, Niccolò Bartolini, Maurizio Zazzi, Ilaria Vicenti, Francesco Saladini

**Affiliations:** Department of Medical Biotechnologies, University of Siena, 53100 Siena, Italy; camilla.biba@student.unisi.it (C.B.); lia.fiaschi@unisi.it (L.F.); ilenia.varasi@student.unisi.it (I.V.); c.paletti@student.unisi.it (C.P.); niccolo.bartolini@student.unisi.it (N.B.); zazzi@unisi.it (M.Z.); saladini6@unisi.it (F.S.)

The authors wish to make the following corrections to this original publication [1]:

In Section 2.1 “Samples”, one sample with a viral load <500 HIV-1 RNA copies/mL (number 5979) was included in the study although the instructions for the use of the AD4SEQ HIV-1 Solution v2 Kit indicate that the kit is certified for in vitro diagnostic use for samples with a viral load >500 HIV-1 RNA copies/mL. The sample has been excluded from the analysis and removed from Table 2 and the Supplementary Data (Tables S1 and S2). All the analysis described in Section 3 “Results” (Sections 3.1–3.4) have been adjusted accordingly where appropriate. The Abstract has been updated accordingly as well as per the overall revisions. Please note that changes to Table 5 occur in the table caption only and no changes have been made to its table content.

In Section 3.2 “Comparison between Homemade NGS and AD4SEQ: Identification of Drug Resistance Mutations” and Section 3.3 “Comparison of SmartVir and HIVdb NGS Data Processing”, the inspection of the SmartVir analysis upstream of the output report revealed that the S68G and V179T mutations were indeed correctly detected but not included in the report itself because they did not contribute to resistance in those samples. Where appropriate, both in the main text (involving Sections 2.6, 3.2–3.4 and 4) and in the supplementary data, the terms or expressions “detected”, “identified”, “considered”, “failed to recognise” and failed to detect” were replaced with “report” or “did not report”. Moreover, the following paragraph has been added to explain this in the “Discussion” Section: “Indeed, inspection of the AD4SEQ raw data clarified that S68G was identified at a frequency comparable with that of the homemade NGS system in all cases. An inquiry with the AD4SEQ manufacturer clarified that S68G was deliberately not included in the report because it does not confer any resistance unless K65R is also present, which was not the case in any of the samples analysed. However, HIVdb reports S68G even when alone because polymorphisms involved in drug resistance may play a role and since clinical use of HIV drug resistance is typically based on cumulative HIV genotype data obtained throughout patient history. The same considerations apply to the RT V179T mutation, which triggers a score for the NNRTI etravirine only in the presence of the Y181C RAM. SmartVir reported V179T in one case together with Y181C but not in the two other cases where it occurred without Y181C”.

The authors state that the scientific conclusions are unaffected and apologise for any inconvenience caused to the readers by these changes.

All corrected paragraphs of sections that involve the above-mentioned changes are as follows:

**Abstract:**

Background: Next-generation sequencing (NGS) kits are needed to finalise the transition from Sanger sequencing to NGS in HIV-1 genotypic drug resistance testing. Materials and Methods: We compared a homemade NGS amplicon-based protocol and the AD4SEQ HIV-1 Solution v2 (AD4SEQ) NGS kit from Arrow Diagnostics for identifying resistance-associated mutations (RAMs) above the 5% threshold in 27 plasma samples where Sanger sequencing previously detected at least one RAM. Results: The samples had a median 4.9 log [IQR 4.6–5.2] HIV-1 RNA copies/mL and were mostly subtype B (59%) and CRF02_AG (15%). Homemade NGS had a lower rate of samples with low-coverage regions (2/27) compared with AD4SEQ (13/27) (*p* < 0.001). Homemade NGS and AD4SEQ identified additional mutations with respect to Sanger sequencing in 12/27 and 9/27 samples, respectively. However, there were two and eight cases where mutations detected by Sanger sequencing were missed by homemade NGS and AD4SEQ-SmartVir, respectively. The discrepancies between NGS and Sanger sequencing resulted in a few minor differences in drug susceptibility interpretation, mostly for NNRTIs. Conclusions: Both the NGS systems identified additional mutations with respect to Sanger sequencing, and the agreement between them was fair. However, AD4SEQ should benefit from technical adjustments allowing higher sequence coverage.

*2.1. Samples*

Access to residual anonymised plasma samples derived from clinical practice was initially obtained through patients’ informed consent as approved by the local Ethics Committee at the University Hospital of Siena. A total of 27 HIV-1 viremic plasma samples with a previous Sanger sequencing-based genotypic resistance test performed at the Microbiology and Virology Unity of the University Hospital of Siena, Italy, were selected. All samples had at least one RAM to protease inhibitors (PIs), nucleoside and non-nucleoside reverse transcriptase inhibitors (NRTIs and NNRTIs) or integrase inhibitors (INIs). The viral load of each sample was available as quantified by the Cobas^®^ 6800 system (Roche Diagnostic, Basel, Switzerland) for routine testing.

*2.6. HIV-1 Subtyping and Drug Resistance Interpretation*

The assignment of HIV-1 subtypes was performed by the COMET HIV-1 tool [11]. NGS data generated by AD4SEQ were analysed both with the dedicated SmartVir software version 1.0.6 provided by Arrow Diagnostics (AD4SEQ-SmartVir) and by the online HIVdb Drug Resistance Database system (AD4SEQ-HIVdb) (Stanford University; https://hivdb.stanford.edu/; accessed on 1 August 2024). NGS data generated by the homemade system were analysed only by HIVdb. The FASTA files obtained from Sanger sequencing were analysed directly, while the FASTQ files obtained from NGS were first converted to CodFreq files and then analysed to detect RAMs. The CodFreq files list the frequency of the mutations of each nucleotide triplet within the sequenced region. For the analysis of FASTQ files, the minimum read depth parameter was set at >100 reads per position with a mutation detection threshold of 5%, both for SmartVir and for HIVdb, independently of whether the position was associated with drug resistance or not. The Stanford HIVdb 9.6 algorithm was used to infer drug susceptibility from the RAMs identified by the two NGS systems and by Sanger sequencing. Mutations were defined as RAMs when associated with a score for at least one drug or included in a combination rule changing the score for at least one drug in the HIVdb 9.6 algorithm. Agreement among the RAMs reported by the different systems was qualitatively defined when identical RAMs were reported.

*3.1. Samples Included in This Study*

All the 27 plasma samples included in this study (Table 2) had detectable viremia with a median viral load of 4.9 log [IQR 4.6–5.2] HIV-1 RNA copies/mL. Subtype B was identified in 16 (59%) cases and CRF02_AG in 4 (15%).

*3.2. Comparison Between Homemade NGS and AD4SEQ: Identification of Drug Resistance Mutations*

The RAMs reported by the two different data processing methods, SmartVir for the data generated by the AD4SEQ HIV-1 Solution v2 Kit, and HIVdb for Sanger and homemade NGS, are listed in Supplementary Table S1. RAMs to PIs, NRTIs, NNRTIs and INIs were detected in 6, 18, 20 and 6 samples, respectively, by at least one method (Table 3). Homemade NGS and AD4SEQ-SmartVir identified additional mutations with respect to Sanger sequencing in 12/27 and 9/27 samples, respectively.

Agreement between the NGS methods and Sanger sequencing for PIs was observed in 24/27 cases (88.9%). In two cases (samples 5826 and 6570), both NGS methods detected additional RAMs with respect to Sanger sequencing. In one sample (6813), homemade NGS was more sensitive in detecting additional RAMs with respect to Sanger sequencing and AD4SEQ-SmartVir.

With NRTIs, complete agreement among the NGS methods and Sanger sequencing was observed in only 18/27 (66.6%) cases. In three cases (samples 5826, 5974 and 7312), homemade NGS-HIVdb reported additional mutations with respect to AD4SEQ-SmartVir and Sanger sequencing, while in one case (sample 7312), AD4SEQ-SmartVir detected an additional mutation with respect to homemade NGS and Sanger sequencing. However, in four cases (samples 6003, 6006, 6436 and 6493), Sanger sequencing and homemade NGS-HIVdb gave comparable results, and AD4SEQ-SmartVir did not report the S68G mutation. Differently from Sanger sequencing and AD4SEQ-SmartVir, homemade NGS failed to identify mutations D67N and K219E in sample 6471. Agreement between the NGS methods with respect to Sanger sequencing was observed only in one case (sample 6835). A strong discordance among the three methods was observed in sample 7312. Indeed, Sanger sequencing detected the D67N, T69G and K219Q mutations, homemade NGS-HIVdb reported mixed populations at codon 67 (D67ΔN), the additional mutation S68G and a deletion at position 69, while AD4SEQ-SmartVir reported a combination of mutations at position D67(N/E) and the T69N/G mutation.

With NNRTIs, similar to NRTIs, 18/27 (66.7%) cases showed complete agreement across the NGS methods and Sanger sequencing. In four cases (samples 5974, 6436, 6493 and 6817), homemade NGS-HIVdb reported additional mutations with respect to AD4SEQ-SmartVir and Sanger sequencing, while in two cases (samples 6669 and 6813), AD4SEQ-SmartVir reported additional mutations with respect to homemade NGS and Sanger sequencing. Additional mutations detected by both NGS methods but not by Sanger sequencing were observed in three cases (samples 6592, 7312 and 7347).

For INIs, complete agreement among all methods was observed in 24/27 cases (88.9%). In one case (sample 6669), both NGS methods detected the minority RAM E157Q, which was not reported by Sanger sequencing. Homemade NGS was more sensitive in detecting the additional minority RAM T97A with respect to Sanger sequencing and AD4SEQ-SmartVir in sample 7347, while AD4SEQ-SmartVir was more sensitive than homemade NGS with sample 6835, detecting L74M.

In conclusion, AD4SEQ-SmartVir did not report one or more RAMs detected by at least one of the other two systems in 1 (3.7%), 7 (25.9%), 4 (14.8%) and 1 (3.7%) samples for PIs, NRTIs, NNRTIs and INIs, respectively, while homemade NGS-HIVdb did not identify one or more RAMs in 0, 2 (7.4%), 2 (7.4%) and 1 (3.7%) samples detected by at least one of the other two systems for PIs, NRTIs, NNRTIs and INIs, respectively. Differently from HIVdb, AD4SEQ-SmartVir did not report the mutation S68G and mutations identified by Sanger sequencing and homemade NGS with a frequency >20% in one case (sample 6817). By contrast, homemade NGS-HIVdb gave a slightly different identification of RAMs at two amino acid positions that were detected as predominant by Sanger sequencing or with frequency >20% by AD4SEQ-SmartVir in sample 7312.

*3.3. Comparison of SmartVir and HIVdb NGS Data Processing*

Notably, SmartVir and HIVdb generated different outputs for read depth, i.e., median read depth vs. max. and min. coverage, respectively (Supplementary Table S2). Other parameters, such as the regions sequenced with a coverage depth of <100 reads per base, were instead reported by both interpretation systems (Table 4). To compare the two NGS data processing methods, we analysed the FASTQ files obtained by AD4SEQ both with SmartVir (AD4SEQ-SmartVir) and with HIVdb (AD4SEQ-HIVdb). Homemade NGS generated low-coverage data in 2/27 samples, both subtype B, in the initial part of the integrase coding region (Table 4). A coverage depth <100 was more frequent both with AD4SEQ-HIVdb (16/27 cases) and with AD4SEQ-SmartVir (12/27) with respect to homemade NGS (2/27) (*p* < 0.001 and *p* = 0.004, respectively) while the difference between AD4SEQ-HIVdb and AD4SEQ-SmartVir was not statistically significant. The median read depth obtained with homemade NGS and with AD4SEQ-HIVdb was comparable (2203 [IQR 1918–8328] reads vs. 4835 [3327–6512] reads, *p* = 0.148). With AD4SEQ-SmartVir, the lower coverage affected six B and six non-B subtypes, mainly at RT codons 14–49 and 260–319 and IN codons 1–75 and 201–288.

Disagreement between RAMs reported by SmartVir and HIVdb when processing AD4SEQ FastQ files was observed in 10 samples (37.0%; Table 5). HIVdb reported one additional PI RAM in one case. For NRTIs, 1 mutation was reported by SmartVir and not by HIVdb, while 8 mutations were reported by HIVdb and not by Smartvir, including S68G in 6/8 cases. For NNRTIs, two mutations at position V179 were reported by HIVdb but not by SmartVir.

**Table 5.** Discordant report of drug resistance mutations between SmartVir and HIVdb interpretation tools from FASTQ files generated by the AD4SEQ sequencing method. Mutations in bold are those reported by only one interpretation tool. The frequency of each mutation is shown in brackets. There were no discordant cases with integrase inhibitor resistance mutations.

*3.4. Prediction of Drug Susceptibility*

Table 6 shows the impact of the RAMs not reported uniformly by the four methods (Sanger sequencing, homemade NGS, AD4SEQ-SmartVir and AD4SEQ-HIVdb) on the prediction of drug susceptibility. Globally, drug susceptibility predictions were fully concordant in 20/27 (74.1%) samples for all the drugs considered, including PIs (Atazanavir, ATV; Lopinavir, LPV; and Darunavir, DRV), NRTIs (Abacavir, ABC; Tenofovir Alafenamide, TAF; and Lamivudine/Emtricitabine, 3TC/FTC), NNRTIs (Doravirine, DOR; Rilpivirine, RPV; Etravirine, ETR; Efavirenz, EFV; and Nevirapine, NVP) and INIs (Dolutegravir, DTG; Bictegravir, BIC; and Cabotegravir, CAB).

Homemade NGS gave discordant results with respect to AD4SEQ-SmartVir in two samples for NRTIs (6471 and 7312) and in one sample each for NNRTIs (6669) and for INIs (6835). AD4SEQ-SmartVir and AD4SEQ-HIVdb gave discordant results in one sample each for PI (ATV and LPV for 6835) and for NRTIs (ABC, TAF and 3TC/FTC for 7312).

Considering Sanger sequencing as a reference, homemade NGS was in agreement with Sanger sequencing for susceptibility predictions against all INIs and PIs but gave discordant predictions in 4/81 (4.9%) cases for NRTIs (6471 and 7312 for one drug and 6835 for two drugs) and in 14/135 (10.4%) cases for NNRTIs (6592 and 7312 for five drugs, 6493 for three drugs and 7347 for one drug). AD4SEQ-SmartVir was discordant with Sanger sequencing in 3/81 (3.7%) cases for NRTIs (7312 for three drugs), in 16/135 (11.9%) cases for NNRTIs (6592 and 7312 for five drugs, 6493 for three drugs, 6669 for two drugs and 7347 for one drug) and in 1/81 (1.2%) cases for INIs (6835). AD4SEQ-HIVdb gave discordant susceptibility predictions in 1/81 (1.2%) cases for PIs and INIs (6835 in both cases), and in 16/135 (11.9%) samples for NNRTIs (6592 and 7312 for five drugs, 6493 for three drugs, 6669 for two drugs and 7347 for one drug). Notably, most of the discrepancies between either NGS method and Sanger sequencing were explained by a lack of detection of minority RAMs (<20%) by Sanger sequencing, namely, in 10 (37.0%), 7 (25.9%) and 9 (33.3%) out of 27 cases with homemade NGS, AD4SEQ-SmartVir and AD4SEQ-HIVdb, respectively.

**4. Discussion**

NGS has redefined genome sequencing techniques, and HIV-1 genotyping has been an early NGS application in the field of infectious diseases with the aim of identifying minority RAMs that cannot be detected by Sanger sequencing [12]. Since NGS is more diversified and complex than Sanger sequencing, standardisation plays a key role in the transition from Sanger sequencing to NGS in clinical settings. Indeed, recent progress in HIV-1 NGS has led to the CE-IVD certification of some commercial systems, including the Sentosa^®^ SQ HIV Genotyping Assay developed by Vela Diagnostics, the AD4SEQ HIV-1 Solution v2 developed by Arrow Diagnostics and the DeepChek^®^ Assay HIV-1 Full PR/RT/INT Drug Resistance developed by ABL Diagnostics.

Due to the limits of the Sentosa^®^ SQ HIV kit—based on the Ion Torrent platform that is more error prone and expensive than the small footprint Illumina platforms [13]—and the slow introduction of the DeepChek^®^ HIV Assay in Italy, the AD4SEQ HIV-1 Solution v2 is currently the most widely used system in Italy. However, to our knowledge, there are no published studies evaluating the AD4SEQ HIV-1 Solution v2 kit in comparison with other NGS-based HIV-1 genotyping techniques or with the reference Sanger sequencing. In this work, we compared Sanger sequencing, AD4SEQ and a homemade amplicon-based NGS protocol to analyse a panel of 27 plasma samples derived from routine analysis. For both NGS methods, we used an Illumina MiSeq platform. The NGS reads generated by the homemade and AD4SEQ systems were processed by the Stanford HIVdb online tool for sequence reads and by the SmartVir system bundled with AD4SEQ, respectively. In addition, AD4SEQ NGS reads were also processed by HIVdb to compare the output of HIVdb and SmartVir on the same raw data (AD4SEQ-HIVdb vs. AD4SEQ-SmartVir). For both the NGS systems and Sanger sequencing, the RAMs detected were interpreted by the HIVdb 9.6 algorithm.

Overall, the Sanger sequencing and NGS results were more concordant for PI and INI than for NRTI and NNRTI RAMs. A higher proportion of additional PI and NRTI RAMs was detected by homemade NGS compared with AD4SEQ-SmartVir. Notably, in 14.8% of the samples, AD4SEQ-SmartVir did not report resistance mutations against NRTIs, which were also detected by Sanger sequencing. Most of these discrepancies were due to the missing S68G in the RT coding region. This mutation was correctly reported when the AD4SEQ data were analysed with the Stanford HIVdb system, indicating that some settings in the SmartVir pipeline likely led to the exclusion of this polymorphic mutation in the final report. Indeed, inspection of the AD4SEQ raw data clarified that S68G was identified at a frequency comparable with that of the homemade NGS system in all cases. An inquiry with the AD4SEQ manufacturer clarified that S68G was deliberately not included in the report because it does not confer any resistance unless K65R is also present, which was not the case in any of the samples analysed. However, HIVdb reports S68G even when alone because polymorphisms involved in drug resistance may play a role and also because clinical use of HIV drug resistance is typically based on cumulative HIV genotype data obtained throughout patient history. The same considerations apply to the RT V179T mutation, which triggers a score for the NNRTI etravirine only in the presence of the Y181C RAM. SmartVir reported V179T in one case together with Y181C but not in the two other cases where it occurred without Y181C.

Most importantly, a lower coverage with respect to homemade NGS was observed for AD4SEQ (samples with a coverage < 100 reads were 2, 12 and 16 for homemade NGS, AD4SEQ-SmartVir and AD4SEQ-HIVdb, respectively). While we were not able to identify any factor associated with low coverage in our dataset, a recently published study based on a larger number of sequence data generated by the AD4SEQ kit showed that non-B subtype and low viremia are associated with low coverage [14]. It must be noted that the apparently lowest coverage with AD4SEQ-HIVdb may have derived from improper handling of the sequence data contributed by AD4SEQ primers, which are proprietary and unknown. Thus, AD4SEQ data processing through pipelines other than SmartVir may not be recommended.

Reassuringly, predictions of drug susceptibility were affected only for NRTIs and NNRTIs in a few cases and mostly derived from the comparison between Sanger sequencing and NGS, which is expected due to the different sensitivity of the methods. Overall, we did not observe any large shift in the prediction of drug susceptibility from a high score to a low score (i.e., from sensitive to resistant or vice versa).

Several published works [12,15–17] have evaluated the performance of homemade NGS systems with respect to Sanger sequencing and obtained results similar to those shown in this study, consistent with the inherently higher sensitivity of NGS in detecting minority RAMs. While participation in external quality assessment programs is a recognised approach for validating homemade NGS methodologies, CE-IVD-approved kits are essential for clinical use. To date, only the Sentosa^®^ SQ HIV kit has been compared with the reference Sanger sequencing [17,18]. Thus, this is the first paper analysing the performance of AD4SEQ HIV-1 Solution v2 kit by Arrow Diagnostics. Although we had the possibility of selecting samples shown to harbour drug resistant viruses by Sanger sequencing, the number of samples was limited and did not allow a wide representation of HIV-1 subtypes. In addition, all samples had >1000 HIV-1 RNA copies/mL; thus, we could not test the threshold of sensitivity for the AD4SEQ amplification method, reported to be at least 500 HIV-1 RNA copies/mL by the manufacturer. For these reasons, further validation experiments on a larger and more heterogeneous sample panel are advisable for completing the assessment of the system. Notwithstanding such limitations, this study conveys relevant information on the performance and caveats of the AD4SEQ system in clinical settings.

**Supplementary Table S1.** Drug resistance mutations considered for the prediction of drug susceptibility in each sample according to the sequencing system and to the data processing method. Mutations in bold are those identified by only one or two sequencing systems. The frequency of each mutation identified by NGS is shown in brackets.

**Sample**

**Sequencing system - Data processing method**

**PIs**

**NRTIs**

**NNRTIs**

**INIs**

**5826**
Sanger-HIVdbV32I, L33F, M46I, I47V, I54M, Q58E, T74TP K70T, M184VNoneNoneHomemade NGS-HIVdbV32I (99%), L33F (99%), M46I (99%), I47V (99%), **I50V (29%)**, I54M (99%), Q58E (99%), T74P (27%)K70T (78%), **V75I (6.5%)**, M184V (96%)NoneNoneAD4SEQ-SmartVirV32I (99%), L33F (99%), M46I (99%), I47V (99%), **I50V (24%)**, I54M (99%), Q58E (99%), T74P (45%)K70T (70%), M184V (99%)NoneNone
**5974**
Sanger-HIVdbNone
**S68G**
K103N, N348INoneHomemade NGS-HIVdbNone
**S68G (99%), K219Q (7.1%)**
K103N (99%), **V106I (10%)**, N348I (99%)NoneAD4SEQ-SmartVirNoneNoneK103N (99%), N348I (99%)None
**6003**
Sanger-HIVdbNone**S68G**, M184VK103N, K238NR263KHomemade NGS-HIVdbNone**S68G (99%)**, M184V (99%)K103N (98%), K238N (99%)R263K (96%)AD4SEQ-SmartVirNoneM184V (98%)K103N (95%), K238N (96%) R263K (98%)
**6006**
Sanger-HIVdbNone
**S68G**
K103N, N348INoneHomemade NGS-HIVdbNone
**S68G (99%)**
K103N (98%), N348I (98%)NoneAD4SEQ-SmartVirNoneNoneK103N (99%), N348I (99%)None
**6084**
Sanger-HIVdbL10F, M46I, T74PD67N, T215CNoneNoneHomemade NGS-HIVdbL10F (98%), M46I (99%), T74P (64%)D67N (92%), T215C (97%)NoneNoneAD4SEQ-SmartVirL10F (98%), M46I (98%), T74P (69%)D67N (98%), T215C (97%)NoneNone
**6092**
Sanger-HIVdbL90MM41ML, M184V, T215TNSYNoneNoneHomemade NGS-HIVdbL90M (98%)M41L (28%), M184V (97%), T215Y (23%)NoneNoneAD4SEQ-SmartVirL90M (99%)M41L (36%), M184V (97%), T215Y (13%)NoneNone
**6107**
Sanger-HIVdbNoneK219NY181CNoneHomemade NGS-HIVdbNoneK219N (99%)Y181C (98%)NoneAD4SEQ-SmartVirNoneK219N (98%)Y181C (99%)None
**6216**
Sanger-HIVdbNoneM41L, T215DNoneNoneHomemade NGS-HIVdbNoneM41L (97%), T215D (98%)NoneNoneAD4SEQ-SmartVirNoneM41L (94%), T215D (90%)NoneNone
**6222**
Sanger-HIVdbNoneNoneV106I, G190ANoneHomemade NGS-HIVdbNoneNoneV106I (91%), G190A (99%)NoneAD4SEQ-SmartVirNoneNoneV106I (92%), G190A (98%)None
**6322**
Sanger-HIVdbNoneNoneE138A, G190GSNoneHomemade NGS-HIVdbNoneNoneE138A (90%), G190S (41%)NoneAD4SEQ-SmartVirNoneNoneE138A (89%), G190S (38%)None
**6363**
Sanger-HIVdbNoneM184VV106A, F227LNoneHomemade NGS-HIVdbNoneM184V (92%)V106A (97%), F227L (93%)NoneAD4SEQ-SmartVirNoneM184V (98%)V106A (97%), F227L (97%)None
**6408**
Sanger-HIVdbL33F, M46L, L90MT215VNoneNoneHomemade NGS-HIVdbL33F (99%), M46L (99%), L90M (98%)T215V (95%)NoneNoneAD4SEQ-SmartVirL33F (98%), M46L (97%), L90M (99%)T215V (98%)NoneNone
**6436**
Sanger-HIVdbNone
**S68G**
K103NNoneHomemade NGS-HIVdbNone
**S68G (85%)**
K103N (98%), **K238T (7.8%), N348I (6.8%)**NoneAD4SEQ-SmartVirNoneNoneK103N (99%)None
**6471**
Sanger-HIVdbNoneM41L, **D67N**, M184V, L210W, T215Y, **K219KE**L100I, K103N, N348INoneHomemade NGS-HIVdbNoneM41L (98%), M184V (98%), L210W (98%), T215Y (95%)L100I (96%), K103N (98%), N348I (98%)NoneAD4SEQ-SmartVirNoneM41L (98%), **D67N (19%)**, M184V (91%), L210W (98%), T215Y (92%), **K219E (9%)**L100I (99%), K103N (97%), N348I (99%)None
**6493**
Sanger-HIVdbNoneD67G, **S68G**, K70R, M184V, T215I, K219EK103N, V108I, K238T, N348INoneHomemade NGS-HIVdbNoneD67G (99%), **S68G (97%)**, K70R (98%) , M184V (98%), T215I (87%), K219E (78%)K103N (98%) , V108I (98%), **V179D (7%), Y181C (34%)**, K238T (98%), N348I (98%)NoneAD4SEQ-SmartVirNoneD67G (98%), K70R (99%), M184V (92%), T215I (94%), K219E (87%) K103N (98%), V108I (99%), **Y181C (26%)**, K238T (97%), N348I (99%)None
**6570**
Sanger-HIVdbNoneM41L, M184MVNoneNoneHomemade NGS-HIVdb
**K20T (14%)**
M41L (44%), M184V (43%)NoneNoneAD4SEQ-SmartVir
**K20T (9.6%)**
M41L (66%), M184V (70%)NoneNone
**6592**
Sanger-HIVdbNoneNoneE138EKNoneHomemade NGS-HIVdbNoneNone**K101E (40%)**, E138K (23%)NoneAD4SEQ-SmartVirNoneNone**K101E (13%)**, E138K (29%)None
**6669**
Sanger-HIVdbNoneNoneK103KN, V106MNoneHomemade NGS-HIVdbNoneNoneK103N (75%), V106M (24%)
**E157Q (7.1%)**
AD4SEQ-SmartVirNoneNoneK103N (45%), V106M (54%), **Y181C (7%)**
**E157Q (11%)**

**6695**
Sanger-HIVdbNoneNoneA98GNoneHomemade NGS-HIVdbNoneNoneA98G (99%)NoneAD4SEQ-SmartVirNoneNoneA98G (90%)None
**6750**
Sanger-HIVdbNone-V75ME138ANoneHomemade NGS-HIVdbNoneV75M (96%)E138A (99%)NoneAD4SEQ-SmartVirNoneV75M (95%)E138A (97%)None
**6762**
Sanger-HIVdbNoneD67N, K219QK103N, V179T, Y181C, H221YNoneHomemade NGS-HIVdbNoneD67N (90%), K219Q (98%)K103N (99%), V179T (T 76%), Y181C (98%), H221Y (99%)NoneAD4SEQ-SmartVirNoneD67N (92%), K219Q (98%)K103N (99%), V179T (T 76%), Y181C (98%), H221Y (98%)None
**6813**
Sanger-HIVdbQ58ENoneE138A, G190A, M230LL74M, G140S, Q148KHomemade NGS-HIVdbQ58E (99%), **G73S (7.3%)**NoneE138A (99%), G190A (99%), M230L (98%)L74M (98%) G140S (99%), Q148K (98%)AD4SEQ-SmartVirQ58E (98%)None**K103N (7%)**, E138A (92%), G190A (95%), M230L (96%)L74M (97%), G140S (97%), Q148K (98%)
**6817**
Sanger-HIVdbNoneNoneK103KN, **V179T**NoneHomemade NGS-HIVdbNoneNoneK103N (67%), **V106I (5.4%), V179T (99%)**NoneAD4SEQ-SmartVirNoneNoneK103N (69%)None
**6835**
Sanger-HIVdbNoneL210W, T215SNoneNoneHomemade NGS-HIVdbNone**M41L (30%)**, L210W (99%), **T215DS (D: 19%, S 79%)**NoneNoneAD4SEQ-SmartVirNone**M41L (19%)**, L210W (98%), **T215DS (D: 25%, S: 72%)**None
**L74M (10%)**

**6880**
Sanger-HIVdbNoneNoneE138GQ95QKHomemade NGS-HIVdbNoneNoneE138G (96%)Q95K (35%)AD4SEQ-SmartVirNoneNoneE138G (97%)Q95K (36%)
**7312**
Sanger-HIVdbNone**D67Δ**, T69G, K219QA98G, V106INoneHomemade NGS-HIVdbNone**D67ΔN (Δ: 8.9%, N: 53%), S68G (52%), T69Δ (93%)**, K219Q (96%)A98G (99%), V106I (94%), **Y181C (55%)**NoneAD4SEQ-SmartVirNone**D67NE (N: 8%, E: 88%), T69NG (N: 6%, G: 89%),** K219Q (96%)A98G (97%), V106I (98%), **Y181C (66%)**None
**7347**
Sanger-HIVdbNoneNoneK101E, Y181C, N348INoneHomemade NGS-HIVdbNoneNoneK101E (99%), **V106I (16%)**, Y181C (99%), N348I (99%)
**T97A (6%)**
AD4SEQ-SmartVirNoneNoneK101E (99%), **V106I (15%)**, Y181C (96%), N348I (96%)NonePIs, protease inhibitors; NRTIs, nucleoside reverse transcriptase inhibitors; NNRTI, non-NRTI; INIs, integrase inhibitors.

**Supplementary Table S2.** Sequence coverage across the protease (PR), reverse transcriptase (RT) and integrase (IN) regions with the different NGS systems. For AD4SEQ-SmartVir, the maximum and minimum number of reads per base and the frequency of bases with coverage >100x are shown, as provided by the SmartVir software. Median read depth per base for the FASTQ files generated by AD4SEQ and by homemade NGS was determined through the Sequence reads (NGS) analysis of the HIVdb program.

**Sample**

**AD4SEQ-SmartVir**

Median read depth*

**PR**

**RT**

**IN**

**Max Coverage**

**Min Coverage**

**Coverage >100x**

**Max Coverage**

**Min Coverage**

**Coverage >100x**

**Max Coverage**

**Min Coverage**

**Coverage >100x**

**ADSEQ**

**Homemade NGS**

**5826**
80964059100%311685891%18057343100%22561918
**5974**
3550017545100%17662133100%93396886%48351946
**6003**
40581982100%167641196%89380151100%18072203
**6006**
3346416481100%18666268100%15735210100%59232175
**6084**
47811086100%202951099100%63086221100%14982097
**6092**
157724764100%21758984100%43239381100%49641058
**6107**
5007925027100%35946436100%64347088%38493057
**6216**
3755818733100%189455796%10982157100%44331816
**6222**
157494751100%431601168100%90812373%49481979
**6322**
2156910953100%32849577100%11598653100%72762136
**6363**
2082662100%267506496%225074070%2421904
**6408**
112832265100%430601280100%55377102100%43351779
**6436**
129415421100%26335133100%512581628100%29662445
**6471**
3195215885100%28161186%4841199100%3984606
**6493**
2944433100%1331832486%256404179100%90185727
**6570**
3046014947100%27198998100%18362883100%91295423
**6592**
3644517723100%19605333100%118581673%617210118
**6669**
66673212100%28367112100%9754364100%340611868
**6695**
33751550100%24663514100%139651225100%19738328
**6750**
47761829100%73220246100%388632677100%596212581
**6762**
5116223967100%529392633100%231773292100%1703415464
**6813**
5145025113100%539512794100%533924391100%236238767
**6817**
2108410307100%29682944100%167801752100%85214934
**6835**
128276098100%15117797100%4286169100%332719445
**6880**
148787027100%15639402100%199403799%65126250
**7312**
200069792100%43735491%26461327100%51022162
**7347**
62311815100%56354136100%102213291100%4403505











*As calculated by HIVdb











## Figures and Tables

**Table 2:** Subtype and viral load of the 27 plasma samples included in this study.

Sample	Subtype	HIV-1 RNA log10 Copies/mL
5826	B	4.3
5974	F1	4.9
6003	CRF02_AG	3.1
6006	F1	6.0
6084	B	4.9
6092	B	4.9
6107	B	4.6
6216	B	4.8
6222	B	6.3
6322	A6	5.4
6363	B	4.8
6408	B	4.9
6436	CRF02_AG	4.8
6471	B	3.7
6493	CRF01_AE	5.1
6570	B	4.1
6592	C	4.5
6669	CRF02_AG	5.0
6695	B	4.7
6750	B	5.3
6762	B	3.1
6813	B	5.0
6817	B	5.4
6835	B	4.3
6880	F1	6.3
7312	G	4.8
7347	CRF02_AG	5.9
