# Supplementary material for: A Comparison of Sanger Sequencing and Amplicon-Based Next Generation Sequencing Approaches for the Detection of HIV-1 Drug Resistance Mutations
Source: Viruses. 2024 Sep 14;16(9):1465. doi: 10.3390/v16091465 (PMC11437444; doi:10.3390/v16091465)
Supplement: Supplementary file 1 [file viruses-16-01465-s001.zip › viruses-3195776-supplementary.pdf]

Supplementary data

Supplementary table S1. Drug resistance mutations identified in each sample according to the sequencing system and to the data processing method. Mutations in bold are those identified by only one or two sequencing systems. The frequency of each mutation identified by NGS is shown in brackets.

Sample	Sequencing system - Data processing method	PIs	NRTIs	NNRTIs	INIs
**5826**	Sanger-HIVdb	V32I, L33F, M46I, I47V, I54M, Q58E, T74TP	K70T, M184V	None	None
	Homemade NGS-HIVdb	V32I (99%), L33F (99%), M46I (99%), I47V (99%), **I50V (29%)**, I54M (99%), Q58E (99%), T74P (27%)	K70T (78%), **V75I (6.5%)**, M184V (96%)	None	None
	AD4SEQ-SmartVir	V32I (99%), L33F (99%), M46I (99%), I47V (99%), **I50V (24%)**, I54M (99%), Q58E (99%), T74P (45%)	K70T (70%), M184V (99%)	None	None
**5974**	Sanger-HIVdb	None	**S68G**	K103N, N348I	None
	Homemade NGS-HIVdb	None	**S68G (99%), K219Q (7.1%)**	K103N (99%), **V106I (10%)**, N348I (99%)	None
	AD4SEQ-SmartVir	None	None	K103N (99%), N348I (99%)	None
**5979**	Sanger-HIVdb	L33F, I84V	**M41L**, D67N, T215Y	K103N, Y181I	T97A, E138K, G140S, Q148H
	Homemade NGS-HIVdb	L33F (98%), **I54T (10%)**, I84V (98%)	**M41L (99%)**, D67N (95%), T215Y (91%)	K103N (97%), Y181I (97%)	T97A (100%), E138K (99%), G140S (99%), Q148H (99%)
	AD4SEQ-SmartVir	L33F (98%), I84V (98%)	D67N (98%), T215Y (88%)	K103N (99%), Y181I (90%)	T97A (94%), E138K (94%), G140S (93%), Q148H (94%)
**6003**	Sanger-HIVdb	None	**S68G**, M184V	K103N, K238N	R263K
	Homemade NGS-HIVdb	None	**S68G (99%)**, M184V (99%)	K103N (98%), K238N (99%)	R263K (96%)
	AD4SEQ-SmartVir	None	M184V (98%)	K103N (95%), K238N (96%)	R263K (98%)
**6006**	Sanger-HIVdb	None	**S68G**	K103N, N348I	None
	Homemade NGS-HIVdb	None	**S68G (99%)**	K103N (98%), N348I (98%)	None
	AD4SEQ-SmartVir	None	None	K103N (99%), N348I (99%)	None
**6084**	Sanger-HIVdb	L10F, M46I, T74P	D67N, T215C	None	None
	Homemade NGS-HIVdb	L10F (98%), M46I (99%), T74P (64%)	D67N (92%), T215C (97%)	None	None
	AD4SEQ-SmartVir	L10F (98%), M46I (98%), T74P (69%)	D67N (98%), T215C (97%)	None	None
**6092**	Sanger-HIVdb	L90M	M41ML, M184V, T215TNSY	None	None

	Homemade NGS-HIVdb	L90M (98%)	M41L (28%), M184V (97%), T215Y (23%)	None	None
	AD4SEQ-SmartVir	L90M (99%)	M41L (36%), M184V (97%), T215Y (13%)	None	None
**6107**	Sanger-HIVdb	None	K219N	Y181C	None
	Homemade NGS-HIVdb	None	K219N (99%)	Y181C (98%)	None
	AD4SEQ-SmartVir	None	K219N (98%)	Y181C (99%)	None
**6216**	Sanger-HIVdb	None	M41L, T215D	None	None
	Homemade NGS-HIVdb	None	M41L (97%), T215D (98%)	None	None
	AD4SEQ-SmartVir	None	M41L (94%), T215D (90%)	None	None
**6222**	Sanger-HIVdb	None	None	V106I, G190A	None
	Homemade NGS-HIVdb	None	None	V106I (91%), G190A (99%)	None
	AD4SEQ-SmartVir	None	None	V106I (92%), G190A (98%)	None
**6322**	Sanger-HIVdb	None	None	E138A, G190GS	None
	Homemade NGS-HIVdb	None	None	E138A (90%), G190S (41%)	None
	AD4SEQ-SmartVir	None	None	E138A (89%), G190S (38%)	None
**6363**	Sanger-HIVdb	None	M184V	V106A, F227L	None
	Homemade NGS-HIVdb	None	M184V (92%)	V106A (97%), F227L (93%)	None
	AD4SEQ-SmartVir	None	M184V (98%)	V106A (97%), F227L (97%)	None
**6408**	Sanger-HIVdb	L33F, M46L, L90M	T215V	None	None
	Homemade NGS-HIVdb	L33F (99%), M46L (99%), L90M (98%)	T215V (95%)	None	None
	AD4SEQ-SmartVir	L33F (98%), M46L (97%), L90M (99%)	T215V (98%)	None	None
**6436**	Sanger-HIVdb	None	**S68G**	K103N	None
	Homemade NGS-HIVdb	None	**S68G (85%)**	K103N (98%), **K238T (7.8%)**, **N348I (6.8%)**	None
	AD4SEQ-SmartVir	None	None	K103N (99%)	None
**6471**	Sanger-HIVdb	None	M41L, **D67N**, M184V, L210W, T215Y, **K219KE**	L100I, K103N, N348I	None
	Homemade NGS-HIVdb	None	M41L (98%), M184V (98%), L210W (98%), T215Y (95%)	L100I (96%), K103N (98%), N348I (98%)	None
	AD4SEQ-SmartVir	None	M41L (98%), **D67N (19%)**, M184V (91%), L210W (98%), T215Y (92%), **K219E (9%)**	L100I (99%), K103N (97%), N348I (99%)	None
**6493**	Sanger-HIVdb	None	D67G, **S68G**, K70R, M184V, **T215I**, K219E	K103N, V108I, K238T, N348I	None
	Homemade NGS-HIVdb	None	D67G (99%), **S68G (97%)** , K70R (98%) , M184V (98%), **T215I (87%)**, K219E (78%)	K103N (98%) , V108I (98%), **V179D (7%), Y181C (34%)**, K238T (98%), N348I (98%)	None

	AD4SEQ-SmartVir	None	D67G (98%), K70R (99%), M184V (92%), K219E (87%)	K103N (98%), V108I (99%), **Y181C (26%)**, K238T (97%), N348I (99%)	None
**6570**	Sanger-HIVdb	None	M41L, M184MV	None	None
	Homemade NGS-HIVdb	**K20T (14%)**	M41L (44%), M184V (43%)	None	None
	AD4SEQ-SmartVir	**K20T (9.6%)**	M41L (66%), M184V (70%)	None	None
**6592**	Sanger-HIVdb	None	None	E138EK	None
	Homemade NGS-HIVdb	None	None	**K101E (40%),** E138K (23%)	None
	AD4SEQ-SmartVir	None	None	**K101E (13%)**, E138K (29%)	None
**6669**	Sanger-HIVdb	None	None	K103KN, V106M	None
	Homemade NGS-HIVdb	None	None	K103N (75%), V106M (24%)	**E157Q (7.1%)**
	AD4SEQ-SmartVir	None	None	K103N (45%), V106M (54%), **Y181C (7%)**	**E157Q (11%)**
**6695**	Sanger-HIVdb	None	None	A98G	None
	Homemade NGS-HIVdb	None	None	A98G (99%)	None
	AD4SEQ-SmartVir	None	None	A98G (90%)	None
**6750**	Sanger-HIVdb	None-	V75M	E138A	None
	Homemade NGS-HIVdb	None	V75M (96%)	E138A (99%)	None
	AD4SEQ-SmartVir	None	V75M (95%)	E138A (97%)	None
**6762**	Sanger-HIVdb	None	D67N, K219Q	K103N, V179T, Y181C, H221Y	None
	Homemade NGS-HIVdb	None	D67N (90%), K219Q (98%)	K103N (99%), V179T (T 76%), Y181C (98%), H221Y (99%)	None
	AD4SEQ-SmartVir	None	D67N (92%), K219Q (98%)	K103N (99%), V179T (T 76%), Y181C (98%), H221Y (98%)	None
**6813**	Sanger-HIVdb	Q58E	None	E138A, G190A, M230L	L74M, G140S, Q148K
	Homemade NGS-HIVdb	Q58E (99%), **G73S (7.3%)**	None	E138A (99%), G190A (99%), M230L (98%)	L74M (98%) G140S (99%), Q148K (98%)
	AD4SEQ-SmartVir	Q58E (98%)	None	**K103N (7%)**, E138A (92%), G190A (95%), M230L (96%)	L74M (97%), G140S (97%), Q148K (98%)
**6817**	Sanger-HIVdb	None	None	K103KN, **V179T**	None
	Homemade NGS-HIVdb	None	None	K103N (67%), **V106I (5.4%)**, **V179T (99%)**	None
	AD4SEQ-SmartVir	None	None	K103N (69%)	None
**6835**	Sanger-HIVdb	None	L210W, T215S	None	None
	Homemade NGS-HIVdb	None	**M41L (30%)**, L210W (99%), **T215DS (D: 19%, S 79%)**	None	None
	AD4SEQ-SmartVir	None	**M41L (19%)**, L210W (98%), **T215DS (D: 25%, S: 72%)**	None	**L74M (10%)**

**6880**	Sanger-HIVdb	None	None	E138G	Q95QK
	Homemade NGS-HIVdb	None	None	E138G (96%)	Q95K (35%)
	AD4SEQ-SmartVir	None	None	E138G (97%)	Q95K (36%)
**7312**	Sanger-HIVdb	None	**D67Δ**, T69G, K219Q	A98G, V106I	None
	Homemade NGS-HIVdb	None	**D67ΔN (Δ: 8.9%, N: 53%)**, **S68G (52%)**, **T69Δ (93%),** K219Q (96%)	A98G (99%), V106I (94%), **Y181C (55%)**	None
	AD4SEQ-SmartVir	None	**D67NE (N: 8%, E: 88%), T69NG (N: 6%, G: 89%),** K219Q (96%)	A98G (97%), V106I (98%), **Y181C (66%)**	None
**7347**	Sanger-HIVdb	None	None	K101E, Y181C, N348I	None
	Homemade NGS-HIVdb	None	None	K101E (99%), **V106I (16%)**, Y181C (99%), N348I (99%)	**T97A (6%)**
	AD4SEQ-SmartVir	None	None	K101E (99%), **V106I (15%)**, Y181C (96%), N348I (96%)	None

PIs, protease inhibitors; NRTIs, nucleoside reverse transcriptase inhibitors; NNRTI, non-NRTI; INIs, integrase inhibitors.

Supplementary table S2. Sequence coverage across the protease (PR), reverse transcriptase (RT) and integrase (IN) regions with the different NGS systems. For AD4SEQ-Smartvir, the maximum and minimum number of reads per base and the frequency of bases with coverage >100x are shown, as provided by the SmartVir software. Median read depth per base for the FASTQ files generated by AD4SEQ and by homemade NGS was determined through the Sequence reads (NGS) analysis of the HIVdb program.

Sample	AD4SEQ-Smartvir									Median read depth*	
	PR			RT			IN				
	Max Coverage	Min Coverage	Coverage >100x	Max Coverage	Min Coverage	Coverage >100x	Max Coverage	Min Coverage	Coverage >100x	ADSEQ	Homemade NGS
**5826**	8096	4059	100%	31168	58	91%	18057	343	100%	2256	1918
**5974**	35500	17545	100%	17662	133	100%	9339	68	86%	4835	1946
**5979**	11706	5770	100%	5770	11	88%	688	1	61%	856	1757
**6003**	4058	1982	100%	16764	11	96%	89380	151	100%	1807	2203
**6006**	33464	16481	100%	18666	268	100%	15735	210	100%	5923	2175
**6084**	4781	1086	100%	20295	1099	100%	63086	221	100%	1498	2097
**6092**	15772	4764	100%	21758	984	100%	43239	381	100%	4964	1058

**6107**	50079	25027	100%	35946	436	100%	6434	70	88%	3849	3057
**6216**	37558	18733	100%	18945	57	96%	10982	157	100%	4433	1816
**6222**	15749	4751	100%	43160	1168	100%	9081	23	73%	4948	1979
**6322**	21569	10953	100%	32849	577	100%	11598	653	100%	7276	2136
**6363**	2082	662	100%	26750	64	96%	22507	40	70%	2421	904
**6408**	11283	2265	100%	43060	1280	100%	55377	102	100%	4335	1779
**6436**	12941	5421	100%	26335	133	100%	51258	1628	100%	2966	2445
**6471**	31952	15885	100%	2816	11	86%	4841	199	100%	3984	606
**6493**	2944	433	100%	133183	24	86%	25640	4179	100%	9018	5727
**6570**	30460	14947	100%	27198	998	100%	18362	883	100%	9129	5423
**6592**	36445	17723	100%	19605	333	100%	11858	16	73%	6172	10118
**6669**	6667	3212	100%	28367	112	100%	9754	364	100%	3406	11868
**6695**	3375	1550	100%	24663	514	100%	13965	1225	100%	1973	8328
**6750**	4776	1829	100%	73220	246	100%	38863	2677	100%	5962	12581
**6762**	51162	23967	100%	52939	2633	100%	23177	3292	100%	17034	15464
**6813**	51450	25113	100%	53951	2794	100%	53392	4391	100%	23623	8767
**6817**	21084	10307	100%	29682	944	100%	16780	1752	100%	8521	4934
**6835**	12827	6098	100%	15117	797	100%	4286	169	100%	3327	19445
**6880**	14878	7027	100%	15639	402	100%	19940	37	99%	6512	6250
**7312**	20006	9792	100%	43735	4	91%	26461	327	100%	5102	2162
**7347**	6231	1815	100%	56354	136	100%	10221	3291	100%	4403	505

*As calculated by HIVdb
